# Genetic Aspects of Scurvy and the European Famine of 1845–1848

**DOI:** 10.3390/nu5093582

**Published:** 2013-09-12

**Authors:** Joris R. Delanghe, Marc L. De Buyzere, Marijn M. Speeckaert, Michel R. Langlois

**Affiliations:** 1 Department of Laboratory Medicine, Ghent University Hospital, B 9000 Ghent, Belgium; 2 Department of Cardiology, Ghent University Hospital, B 9000 Ghent, Belgium; E-Mail: marc.debuyzere@ugent.be; 3 Department of Nephrology, Ghent University Hospital, B 9000 Ghent, Belgium; E-Mail: marijn.speeckaert@ugent.be; 4 Department of Laboratory Medicine, AZ Sint Jan Hospital, B 8000 Bruges, Belgium; E-Mail: Michel.Langlois@azsintjan.be

**Keywords:** haptoglobin, iron, polymorphism, scurvy, vitamin C

## Abstract

The view of scurvy being exclusively a nutritional disorder needs to be updated. Genetic polymorphisms of *HFE* and haptoglobin (Hp) may explain the geographic variability of mortality caused by the European famine of the mid-19th century. In this period, potatoes had fallen victim to the potato blight and Ireland was more severely hit than continental Europe. Hereditary hemochromatosis is a genetic disorder with mutations in the *HFE* gene, characterized by iron overload (with a reduced vitamin C stability) and with a predominance of affected men. The Irish have the world’s highest frequency of the C282Y mutation and the particular iron metabolism of the Irish helps to understand the size of the catastrophe and the observed overrepresentation of male skeletons showing scurvy. Hp is a plasma α_2_-glycoprotein characterized by 3 common phenotypes (Hp 1-1, Hp 2-1 and Hp 2-2). When the antioxidant capacity of Hp is insufficient, its role is taken over by hemopexin and vitamin C. The relative number of scurvy victims corresponds with the Hp 2-2 frequency, which is associated with iron conservation and has an impact on vitamin C stability. As iron is more abundant in males, males are overrepresented in the group of skeletons showing scurvy signs.

## 1. Introduction

In the mid-19th century, Ireland’s potatoes had fallen victim to the potato blight (*phytophthora infestans*). However, the epidemics of the potato late blight in this period were not restricted to Ireland. Also in continental Europe, the potato blight caused the Continental Famine (1844–1846) and—in the longer term—contributed to the European revolutions of 1848. However, it cannot be denied that Ireland was more severely hit than nations of continental Europe [1]. Since potatoes were the main source of vitamin C in the diet, scurvy is generally explained by a shortage of potatoes. However, the relative decline in potato harvest in 1845 in continental Europe was higher than the decline in Ireland [2]. On average, the human body loses ±3% of its vitamin C content per day, which severely limits the disease-free and survival time when subjects are on a diet poor in vitamin C [3]. Although scurvy is classified as a nutritional disorder or avitaminosis, only about 17% of the variance of the serum vitamin C concentration in humans can be explained by vitamin C intake [4]. The vitamin C status is not only determined by diet, but also by environment, lifestyle, biological and pathological conditions [5,6]. The present paper wants to highlight the impact of the particular genetic effects, which helps to understand the geographical distribution of the European mid-19th century famine.

## 2. Why Was Ireland Struck More than the Continent?

Geber and Murphy reported on the pronounced gender difference during the mid-19th century scurvy epidemic [7]. This finding is remarkable since a shared vitamin C poor diet is assumed. In order to better understand the severity of the scurvy outbreak, one should take into account the particular population genetics of the Irish. Hemochromatosis is a disease characterized by iron overload which has been associated with the C282Y (and H63D) mutation. This mutation has probably arisen in either a Celtic or a Viking ancestor some 60 generations ago [8,9]. Clinical symptoms of hemochromatosis show a marked gender difference predominantly affecting men. Moreover, clinical manifestations occur at an earlier age in men. While the Scandinavians have a high C282Y mutation frequency, the Irish have the highest frequency (around 11%) of the C282Y mutation in the world. The hemochromatosis associated iron overload reduces vitamin C stability *in vivo*, which explains the Irish population to be more prone to scurvy than other Europeans in times of reduced vitamin C supply. The particular iron metabolism of the Irish helps to understand the size of the scurvy catastrophe and the observed overrepresentation of male skeletons showing scurvy lesions. However, analyzing those data, we have to take into account that diagnostic traits of disease in the human skeleton normally only occur in its chronic and advanced stage. The prevalence of skeletal indicators of disease is therefore not equal to the true prevalence of the disease, as deceased individuals without any evidence of disease may very well have suffered and died as a consequence of it before it manifested itself skeletally. This fundamental understanding of the representation of disease in skeletal populations is usually referred to as “the osteological paradox” [10]: the healthiest and strongest individuals in a population are likely to be those that developed the worst and most substantial degree of pathological lesions, as they were strong enough to survive long enough for it to be manifested in their skeletal tissues. This is a limitation of using palaeopathological data from archaeological populations in direct analogy with modern clinical data.

The proportion of observed scurvy cases in mid-19th century Ireland is higher in comparison with some well-known documented historical scurvy outbreaks among Europeans. Generally, scurvy outbreaks affect 15%–30% of the European population: the French expedition of Cartier in 1536 counted 25 victims (a rate of ±30%). For the Dutch East Indies Company, typical crew losses for a one-way trip between Europe and the Indonesian archipelago in the 17th century were ±20%. The Dutch expedition to Novaya Zemlya in 1596 counted only 2 scurvy victims among 17 crew members. In the Crimean War (1854–1856), the French Navy counted ±30% of scurvy cases. In Perth general prison in Scotland, scurvy occurred at a rate of 50 of 330 inmates during 1845–1846. In 1819, 160 scurvy victims were counted of 800 soldiers in the US Army outpost at Fort Atkinson (Nebraska) [5,11]. Despite the fact that conditions are not completely comparable, these data are striking because they were recorded in relative homogeneous populations sharing a uniform diet. The prevalence of the haptoglobin (Hp) 2-2 phenotype may partly explain the findings described above, as the relative number of scurvy victims roughly corresponds with the relative frequency of the Hp 2-2 phenotype [12].

Human Hp is a plasma α_2_-glycoprotein characterized by 3 common phenotypes (Hp 1-1, Hp 2-1 and Hp 2-2). Its free hemoglobin (Hb)-binding capacity prevents Hb-driven oxidative damage. When the antioxidant capacity of Hp is insufficient, its role is taken over by hemopexin (heme-binding protein) and by vitamin C (free radical scavenger) [13]. Hp phenotypes show important structural and functional differences, which offer a plausible explanation how during the course of human history, some populations characterized by a high Hp 1 allele frequency have been able to survive on a vitamin C poor diet [6]. In comparison with the other Hp phenotypes, Hp 2-2 is associated with a better iron conservation (characterized by a higher serum iron concentration, higher serum ferritin concentrations and increased transferrin saturation levels) which may act synergistic in presence of hemochromatosis [14,15]. However, the Hp 2-2 phenotype has also a major impact on vitamin C stability *in vivo* [13]. Hp 2-2 subjects are less efficient in removing free Hb from plasma, which may favour an iron-mediated vitamin C depletion [13,16]. Iron retention in scurvy-prone Hp 2-2 individuals results in iron-driven oxidative stress, which is reflected by lower serum vitamin C concentrations in healthy Hp 2-2 subjects [3].

Furthermore, the iron delocalization pathway, selectively occurring in Hp 2-2 subjects has consequences in infections. Iron withholding is an important example of nutritional immunity in the defense against infectious diseases [17]. Hp acts as a natural bacteriostat by preventing the utilization of Hb by pathogenic bacteria which require iron for their growth. Hp polymorphism plays a role in a number of bacterial and viral infections [18]. As Hp 2-2 subjects are more prone to e.g., tuberculosis, a relationship between susceptibility for scurvy and the presence of life threatening infections may be postulated. In an Irish population, prevalence of the Hp 2-2 phenotype is ±41% [19], which roughly corresponds to the proportion of skeletons showing scurvy signs [7]. As iron is more abundant in males than in females, it is not surprising that males are overrepresented in the group of skeletons showing scurvy signs.

## 3. The Situation on the European Continent

Despite the fact that the potato blight also struck continental Europe and the unfavourable climatological circumstances (temperature, humidity) between 1845 and 1847 were comparable among Western European countries, continental Europe was less affected than Ireland. When plotting the C282Y allele frequencies of the various European countries (Belgium, The Netherlands, France, Switzerland, Germany) against the excess mortality of the years 1845–1847 and the decline in potato yields, a good correlation (*y* (excess mortality per million) = −32.42 + 9.22*x* (C282Y allele frequency, %), *r*^2^ = 0.98) was obtained (Table 1). As the Hp phenotype frequency shows more limited differences among European countries [11], no significant correlation between the Hp 2 allele frequency and the excess in mortality was found.

**Figure 1:**
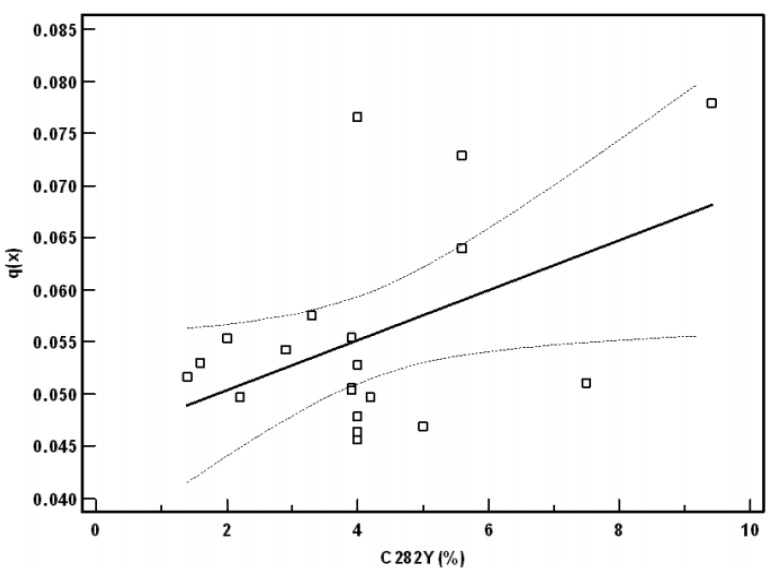
Mortality in 30–35 year old subjects in 1846 according to C282Y allele frequencies in a number of French departments. The *Y*-axis depicts the death probabilities q (*x*) for every age (*x*) computed for that period. The *X*-axis represents the C282Y allele frequency in the different departments.

Also in France, a country with important Celtic influences, a marked correlation between the C282Y mutation frequency and mortality was observed for the various departments. When comparing the 1846 mortality among the population aged 30–35 years with the C282Y allele frequency for the various departments (Figure 1), a marked correlation was found, which resembles the one found for Western Europe. Higher C282Y frequencies were associated with higher mortality rates. Both in the 1841 and 1851 French census [20], the effect of the C282Y allele frequency on mortality of 30 year old subjects was greatly reduced, as evidenced by the regression coefficients (1841:0.016; 1846:0.024; 1851:0.016).

**Table 1:** Overview of annual excess mortality, decline in potato yields and C282Y mutation frequency in various European countries.

Country	Annual Excess Mortality [1846–1848 (per million)] [1]	Decline in Potato Yields (1845) [2]	C282Y Mutation Frequency [9]
Austria	7	−87% −20% −55% −30% −71%	4.1%
Belgium	3		4.1%
France	1		4%
Germany	2		2.6%
Ireland	100		14.2%
The Netherlands	3		4.1%
Switzerland	0		4.1%

## 4. Conclusions

In conclusion, the classical view of vitamin C deficiency and scurvy being exclusively nutritional disorders needs to be updated. The genetic polymorphism of *HFE* and (to a lesser extent) Hp may have played an important role in explaining the remarkable geographic variability of excess in mortality during famine and scurvy episodes in recent European history. The data confirm the importance of the interplay between the iron status and vitamin C requirements. In order to further elucidate the mechanisms of scurvy, *HFE* and Hp genotyping of skeletons of scurvy victims could be considered in the future. Besides the differential genetic background, cultural, social and economic aspects relating to access to food and differential diets in the 19th century should be taken into account when interpreting those results.
